# The Association between Sleep Patterns, Educational Identity, and School Performance in Adolescents

**DOI:** 10.3390/brainsci13020178

**Published:** 2023-01-20

**Authors:** Valeria Bacaro, Alice Andreose, Martina Grimaldi, Vincenzo Natale, Lorenzo Tonetti, Elisabetta Crocetti

**Affiliations:** Department of Psychology “Renzo Canestrari”, Alma Mater Studiorum University of Bologna, Viale Berti Pichat 5, 40126 Bologna, Italy

**Keywords:** adolescents, sleep, social jetlag, catch-up sleep, school performance, school experience

## Abstract

Adolescents’ school experience can be developmentally related to adolescents’ sleep. This study aimed to understand how sleep patterns (i.e., sleep duration and sleep-schedule) and weekend sleep-recovery strategies (i.e., social jetlag and weekend catch-up sleep) are associated with adolescents’ school experience (i.e., educational identity and school performance). Moreover, the differences in the school experiences between adolescents with different numbers of weekend-sleep-recovery strategies were assessed. For this purpose, 542 Italian adolescents (55.2% females, mean age 15.6 years) wore an actigraph for one week. After the actigraphic assessment, questionnaires on educational identity and school performance were administered. Results showed that short sleep-duration, later bedtime during weekdays and weekends, and a higher amount of social jetlag were negatively associated with school performance. Furthermore, adolescents who did not use any sleep-recovery strategy during the weekend presented lower levels of educational in-depth exploration compared to adolescents with higher levels of catch-up sleep but not social jetlag. These data pointed out a potentially detrimental role of social jetlag on school performance and differences in identity processes between adolescents who used and those who did not use sleep-recovery strategies, which could affect adolescents’ psychosocial adjustment.

## 1. Introduction

Sleep is a crucial psychophysiological process influencing the health and development of adolescents [1,2]. At the same time, adolescents face changes in cognitive, behavioral, and emotional functioning, along with significant changes in their social relationships that could affect their sleep patterns [3]. The perfect-storm model [4] underlined an interplay between bioregulatory mechanisms and psychosocial factors that may lead to a deficient environment for adolescents’ sleep (e.g., late-night screen time, newfound autonomy in choosing their bedtime, early school-start times, social engagement, and caffeine intake). This is in line with previous reports, which highlighted the fact that nowadays, most adolescents are sleep-deprived, compared to the National Sleep Foundation suggestions, which indicated a range of between 8 and 10 h as the proper amount of sleep duration for this age group [5,6]. This has an increased impact, and is being recognized as a public health issue [7], since sleep deprivation is linked to many adverse health outcomes involving cognitive, physical, and mental-health problems [8,9].

One of the main factors that could contribute to the sleep deficit in adolescents is constituted by their social schedules. Indeed, irregular sleep-patterns, activity schedules, and the shift in circadian preference from morning- to evening-type may result in sleep deprivation or sleep deficit in adolescents [10]. Specifically, adolescents’ weekend and weekday sleep-schedules differ significantly, due to school attendance [11]. This misalignment of social and biological times impacts not only the sleep duration but also the regularity of adolescents’ sleep-schedule. Thus, one of the main strategies adolescents adopt to deal with this discrepancy, and consequent sleep-debt, is to delay the midpoint of sleep during weekends, defined as social jetlag [12]. Social jetlag has been identified as a potential risk-factor for psychological and physical well-being [13,14]. Furthermore, another sleep-recovery strategy that adolescents could use consists of extending weekend sleep-duration (i.e., weekend catch-up sleep). On the one hand, previous results showed that catch-up sleep could have beneficial effects on the metabolism and psychological well-being, since it may help to diminish the risks associated with cumulative sleep-deprivation during the weekdays [15]. On the other hand, it was also reported that the consequences of weekend catch-up sleep could depend on its duration. Larger amounts of weekend catch-up sleep could be associated with a higher level of sleep deprivation during the weekdays, and circadian misalignment, leading to detrimental effects on adolescents’ mental health [16]. Nevertheless, information on the association between weekend catch-up sleep and adaption in adolescents is currently limited, and it is of utmost importance to understand how the combination of these two strategies (i.e., social jetlag and weekend catch-up sleep) can affect the diverse aspects of adolescents’ everyday life [17].

The school context has a pervasive influence on the daily-life experiences of adolescents. One of the key tasks adolescents face is to develop their educational identity [18,19]. Educational identity refers to the goals and choices adolescents delineate and consider, related to their educational context [20]. According to the three-factor identity model [21,22], adolescents can develop their educational identity through a dynamic and iterative process, based on the interplay among commitment, in-depth exploration, and reconsideration of commitment. Specifically, commitment refers to enduring choices individuals have made about their education and to the self-confidence they derive from these choices. In-depth exploration indicates the extent to which individuals think actively about the educational commitments they have made, reflecting on their choices, searching for additional information, and talking with others about their commitments. Reconsideration of commitment refers to comparing current educational-commitments with possible alternatives because the current ones are no longer satisfactory [23]. Prior research highlighted the fact that each identity process is related to a host of indicators of psychosocial adjustment [for a review, see 22]. Nonetheless, no studies evaluated how adolescents’ sleep patterns and sleep-recovery strategies during the weekend could be related to educational-identity development.

Moreover, adolescents’ educational identity is intricately linked to their school performance [24]. In this vein, school performance (i.e., the measure of students’ success or failure) represents the feedback adolescents receive from the school environment, and can drive changes in educational identity. Previous research showed how higher levels of school performance led to high levels of commitment, while low levels of school performance led to higher levels of reconsideration of commitment [25]. In this picture, sleep patterns may play a significant role. Consistent evidence has indicated how sleep difficulties and short sleep-duration could be associated with poor school performance; for a review, see [26]. This phenomenon can be explained through several mechanisms, such as increased daytime sleepiness due to sleep-debt [27]. Nevertheless, results showed low correlations related to the link between poor sleep-duration and quality, and school performance. That could be due to the fact that the evaluation of different sleep domains does not account for individuals’ sleep need and vulnerability to sleep loss.

In line with this reasoning, the current study aims to examine the association of objectively measured sleep patterns (considering both weekday and weekend) and weekend sleep-recovery strategies (taking into account both social jetlag and catch-up sleep) with different aspects of the school experience (i.e., educational-identity processes and school performance) in adolescents. In particular, the first aim is to understand which aspects of the weekday and weekend sleep-patterns and weekend sleep-recovery strategies are associated with educational identity and school performance. The second aim is to examine the differences in the school experiences among adolescents with different amounts of weekend sleep-recovery strategies. Therefore, it will be possible to better understand the potential protective or detrimental role of the different weekend sleep-recovery strategies for adolescents’ school experience and psychosocial adjustment. Moreover, it could also be possible to understand which aspects of adolescents’ sleep patterns could be more adaptive, and promote the implementation of evidence-based interventions in the school context.

## 2. Materials and Methods

### 2.1. Participants

An overall number of 542 participants (55.2% females), with a mean age of 15.6 years (standard deviation = 1.2 years, range = 14.0–20.1 years), were examined in the current study. Participants attended either the first year (50.4%) or the third year (49.6%) of secondary high-school. Most of the adolescents attended academic schools (43%), and the remaining were balanced between technical (18.2%), professional institutes (17%), or mixed-type schools (21.8%).

### 2.2. Procedure

The present study was approved by the Bioethics Committee of the Alma Mater Studiorum University of Bologna (Italy) (Prot. n. 263836 of 14/10/2021) as part of the ERC-Consolidator project IDENTITIES “Managing identities in diverse societies: a developmental intergroup perspective with adolescents”. This study is part of a larger longitudinal research study involving 1st- and 3rd-year students (at the first assessment) from several high schools in the northern part of Italy (i.e., the Emilia-Romagna region), their parents, and teachers. For this study purpose, participants were included in the final database for statistical analyses only if, at the actigraphic recording, they had at least three valid nights during school days and one valid night during the weekend. No other exclusion criteria were applied for the final sample of this study.

Schools were selected through a stratified (by school-tracking and level of urbanization of the area) randomized method, and principals were approached to present the project. Upon their approval, the study was then presented to students and their parents, who also received written and detailed information about it. Parents’ active consent was obtained. Active consent was also obtained from adolescents who were of age while their underaged peers provided their assent to participate in the project. Participation in the study was voluntary, and students were informed that they could withdraw their consent at any time.

The ongoing longitudinal project started in 2022, and includes multiple annual, monthly, and daily assessments. Data from the second wave (April/May 2022) has been used for the present study. All the involved participants were invited to wear an actigraph Micro MotionloggerWatch (Ambulatory Monitoring, Inc., Arsdely, NY, USA) for one week. After the actigraphic assessment, questionnaires on subjective sleep–wake quality and psychosocial variables were administered during school hours.

### 2.3. Instrument

**Objective sleep-parameters.** In order to evaluate objective sleep-quality and parameters, adolescents wore a wrist actigraph (Micro Motionlogger Watch, Ambulatory Monitoring, Inc.; Ardsley, NY) for seven consecutive nights. The actigraph was initialized through the Motionlogger Watchware software (Ambulatory Monitoring, Inc., Ardsley, NY, USA) in zero-crossing mode to collect data in 1-min epochs. Adolescents were instructed to wear the actigraph for one week, and to press the event marker on the actigraph to indicate when they (a) turned off the lights to go to sleep at night, and (b) got out of bed in the morning. The actigraphic data were analyzed through Action W-2^®^ software, version 2.7.1 (Ambulatory Monitoring, Inc., Ardsley, NY). This software identified each epoch as sleep or wake, using the mathematical model validated by Cole and Kripke [28] and Cole and colleagues [29]. In the present study, we considered the following actigraphic sleep-parameters: time in bed (i.e., defined as the interval between bedtime and get-up time); the midpoint of sleep (i.e., a sleep-phase marker expressed in hours and minutes, which splits the time in bed in half); bedtime; get-up time; and total sleep time in minutes (i.e., the sum of all sleep epochs between sleep start, with the first epoch of the first 20-min block of persistent sleep, with no more than 1 min of wake). Furthermore, social jetlag was computed as the difference in the midpoint of sleep recorded during the weekend and school days. The length of the social jetlag was expressed in minutes, with positive values pointing to a delayed midpoint of sleep during the weekend days compared to the school days. The weekend catch-up sleep was calculated as the difference in the total sleep-time between weekend days and weekdays. Therefore, a positive value means a higher duration of total sleep-time during the weekend days, compared to the school days. According to the aims of this study, participants were then split into four groups, based on the amount of social jetlag (substantial amount of social jetlag: ≥90 min) and/or weekend catch-up sleep (substantial amount of catch-up sleep: ≥60 min) that they implemented. These categories were based on previous classification [17], according to the criteria of the frequency distribution, keeping as a reference the quartiles (regarding social jetlag, 46.9% of adolescents reported less than 90 min of social jetlag, and regarding catch-up sleep, 49% reported less than 60 min of catch-up sleep). Specifically, the four groups were: (1) participants with a small amount of both social jetlag and catch-up sleep (*n =* 142 ; 26.2%); (2) participants with a large amount of catch-up sleep but not social jetlag (*n =* 110 ; 22.9%); (3) participants with a large amount of social jetlag but not catch-up sleep (*n =* 124 ; 20.3%), and (4) participants with a large amount of social jetlag and a large amount of catch-up sleep (*n =* 166 ; 30.6%). A Table presenting descriptive data of the respondents in these four groups is available in the Supplementary Materials Table S1.

**Educational identity processes.** Commitment, in-depth exploration, and reconsideration of commitment in the educational domain were measured using the Utrecht-Management of Identity Commitments Scale (U-MICS) [21,30]. It consists of 13 items rated on a Likert-type rating scale (from 1 “Completely false” to 5 “Completely true”). Sample items include “My education gives me certainty in life” (commitment; 5 items), “I think a lot about my education” (in-depth exploration; 5 items), and “I often think it would be better to try to find a different education” (reconsideration of commitment; 3 items). Cronbach’s alphas were 0.88 for commitment, 0.75 for in-depth exploration, and 0.81 for reconsideration of commitment.

**School performance.** This was assessed by asking adolescents to self-report their grade-point average (GPA). The Italian grading system uses a 10-point scale, with 1 representing the minimum and 10 being the maximum grade (a grade of 6 is the minimum accepted requirement to be positively evaluated).

### 2.4. Strategy of Analysis

The mean scores of the filled-in questionnaires and the means of the actigraphic sleep-parameters recorded during the week of registration were used for data analysis. Pearson’s correlations analyses were performed to examine the associations between sleep parameters (i.e., the duration of total sleep-time during weekdays and weekends, bedtime and get-up time during weekdays and weekends, social jetlag, and weekend catch-up sleep) and school experiences (i.e., educational-identity processes and school performance). Finally, one-way analysis of variance (ANOVA) was performed with the social- jetlag and weekend-catch-up-sleep groups as the between-subjects factor, and school- experience variables as the dependent variables. In the case of significant effects, the HSD Tukey post-hoc test was used, with the significance level set to *p* < 0.05. All statistical analyses were performed using IBM SPSS Statistics version 28.0.

## 3. Results

### 3.1. Descriptive Statistics

As shown in Table 1, concerning objectively measured sleep-characteristics of the sample, adolescents reported, on average, a late bedtime both on weekdays and weekends (after 23:00) and an amount of social jetlag and weekend catch-up sleep of more than 1 h. Moreover, adolescents reported medium levels of educational-identity processes and a medium level of average school- performance.

### 3.2. Associations between Objective Sleep-Parameters and School Experience

Correlations between the total amount of social jetlag and weekend catch-up sleep, sleep duration and bedtime during the weekends and weekdays, and educational-identity processes and school performance are reported in Table 2. As can be seen, results showed that short sleep-duration, later bedtime during weekdays and weekends, and higher amount of social jetlag were negatively associated with school performance. Finally, a later bedtime during the week was associated with lower levels of educational commitment.

### 3.3. Differences between the Weekend-Sleep-Recovery-Strategies Groups

The one-way analysis of variance (ANOVA) with groups based on the amount of social jetlag and weekend catch-up sleep as the independent variable and school-experience variables as the dependent variables showed a significant difference only in educational in-depth exploration (Table 3). Tukey post hoc comparisons highlighted the fact that adolescents without a substantial amount of both sleep-recovery strategies had significantly lower levels of in depth-exploration compared to the group of adolescents with a high amount of catch-up sleep but not social jetlag. Partial-eta-squared values indicated that the effect size was small.

## 4. Discussion

The current study aimed to examine the potential association between objectively measured sleep-patterns during the week and weekend, sleep-recovery strategies during the weekend spontaneously implemented by adolescents, and educational identity and school performance. Specifically, concerning the first aim, results showed that a longer duration of total sleep-time and earlier bedtime during the weekdays and weekends were linked with a higher level of school performance. Moreover, later bedtime during the weekdays was associated with lower levels of educational commitment. Concerning sleep-recovery strategies, a higher amount of social jetlag was found to be associated with lower school-performance. Regarding the second aim, results showed that adolescents without higher amounts of weekend sleep-recovery strategies reported a lower level of in-depth exploration compared to adolescents with a higher level of catch-up sleep but not social jetlag.

These findings highlighted the potential association between adolescents’ regular sleep-patterns and duration, and their school experience. In particular, these results align with previous findings that highlighted the association between short sleep-duration and irregular sleep-schedules with poorer school-performance [26]. Short sleep-duration and later bedtimes were found to be associated with a plethora of negative outcomes in several crucial spheres of adolescents’ lives (e.g., unhealthy behavior, depressive symptoms, emotional dysregulation, and diurnal-functioning impairment) [13,14,31] that can also influence the adolescents’ school performance [32]. Concurrently, societal constraints, bioregulatory changes, and psychosocial factors (e.g., increase in school and social-relationships demands; additional light-stimuli) can sustain evening alertness later into the night, potentially reinforcing the perpetuation of late bedtime and short sleep-duration of adolescents [33]. Another aspect that was found to be associated with poorer school-performance was the amount of social jetlag, and this could be due to the effects of sleep-debt accumulated on weekdays and consequent impairment in daytime functioning [29]. These findings align with previous studies that underline the important potential role of social jetlag in worsening school-performance [34], and call for future longitudinal studies to understand the long-term effect of this sleep-recovery strategy. Thus, on the one hand, sleep-recovery strategies could help compensate for adolescents’ sleep-debt during the week in the short term; on the other hand, the misalignment of sleep-schedule and prolonged sleep-duration could be linked to adolescents’ health and psychosocial adjustment.

Furthermore, another interesting result that emerged from this study was that adolescents who did not implement any sleep-recovery strategy during the weekend presented lower levels of in-depth exploration, compared to the group with a large amount of catch-up sleep but not social jetlag. In-depth exploration has been conceptualized as a sort of double-edged sword, linked to both positive (e.g., openness to experience, social responsibility) [21,35] and negative outcomes (e.g., lower self-esteem, higher symptoms of distress and anxiety) [36,37], which arise when individuals might start to doubt their current commitments. The results of this study add to this evidence by showing that a higher level of in depth-exploration was present in adolescents with a higher level of sleep compensation during the weekend, compared to adolescents who did not report a large number of sleep-recovery strategies. These findings can be interpreted by considering that in-depth exploration is a demanding process, as adolescents are actively involved in considering the meaning of their educational commitments. Adolescents with large amount of catch-up sleep but no social jetlag tended to maintain a routine in terms of bedtime and get-up time, not shifting their midpoint of more than or equal to 90 min during the weekend compared to the weekdays, but they compensate their slept-debt with longer sleep-duration during the weekend, compared to the school days. Thus, it is probable that adolescents are required to use some cognitive strategies to compensate for their sleep-debts.

This study has some limitations. First, cross-sectional data were presented based on only one week of registration. As the longitudinal project in which this study is embedded continues, it will be possible to better understand the strength and direction of these effects in the short, medium, and long term. Another limitation is referring to the measurement of school performance, which was assessed through adolescents’ self-reported average grades. Although self-reported grades are highly correlated with actual high-school GPA (*r* = 0.82) [38], future studies could benefit from replicating the results by relying on school records to obtain a more objective estimate of adolescents’ school performance. Furthermore, this study did not consider the role of adolescents’ chronotypes [10]. Future studies should consider the role of chronotype in adolescents, since social jetlag and chronic sleep-debt are particularly present in individuals with a more evening chronotype, as the standard pattern of early school-start times increases the misalignment with biological rhythms [39]. Finally, this study did not exclude adolescents with mental or neurological pathologies that could potentially affect their sleep pattern. Future studies should address this gap by evaluating sleep patterns and the impact of sleep-recovery strategies also in clinical populations. This could provide novel insights into identifying the population particularly at risk and in implementing prevention-targeted programs, to ameliorate their sleep patterns. Moreover, other adolescents’ typical at-risk behaviors, such as substance use and digital-media overuse [40,41], which can negatively impact their sleep, and subjective school experience should be controlled when evaluating this association. Despite its limitations, the present study underlined the importance of considering the potential role of weekday and weekend sleep-patterns and sleep-recovery strategies and their connection with adolescents’ psychosocial adjustment. Moreover, results also suggested the importance of examining the long-term effect of adolescents’ protracted sleep-duration and irregular sleep-schedule, together with the weekend sleep-recovery strategies they implement to compensate for their sleep-debt. In particular, since during this life stage, adolescents experience a shift toward eveningness, also due to their changes in relational and social spheres, such as school demands, new social opportunities, and less parental supervision, it is of utmost importance to understand which strategy could be more protective for their school experience. In this way, it will be possible to develop and implement psychoeducational, targeted, and evidence-based interventions to keep teachers, families, and adolescents aware of the potentially detrimental effect of their sleep-debt, starting with changing policies and suggesting healthy behaviors in their most pervasive social contexts (i.e., family and school).

## Figures and Tables

**Table 1:** Sleep characteristics of the sample. Means (M) and standard deviations (SD) are shown.

Sleep Variables	M	SD
Amount of social jetlag (in minutes)	100.69	66.59
Amount of catch-up sleep (in minutes)	63.64	95.16
Total sleep-time during the week (in minutes)	405.07	49.21
Total sleep-time during the weekend (in minutes)	468.79	89.89
Bedtime during the week	23:26	0.84
Bedtime during the weekend	24:31	1.40
Get-up time during the week	6:45	0.60
Get-up time during the weekend	9:35	1.49
School-experience Variables	M	SD
Educational Commitment	3.25	0.79
Educational in-depth exploration	3.22	0.69
Educational reconsideration of commitment	2.97	0.90
School performance	7.30	0.89

**Table 2:** Correlations between objective sleep-patterns and school-experience variables. Significant correlations are marked with asterisk(s).

Variables	1	2	3	4	5	6	7	8	9	10
1. Social jetlag (in hours)	-									
2. Weekend catch-up sleep (in minutes)	0.09 *	-								
3. Total sleep-time during the weekend (in minutes)	0.04	0.86 ***	-							
4. Total sleep-time during the week (in minutes)	−0.10 *	−0.36 ***	0.16 ***	-						
5. Bedtime during the weekend	0.75 ***	−0.27 ***	−0.44 ***	−0.27 ***	-					
6. Bedtime during the week	0.03	0.36 ***	0.01	−0.67 **	0.40 ***	-				
7. Educational commitment	−0.01	0.05	0.07	0.03	−0.08	−0.09 *	-			
8. Educational in-depth exploration	0.01	0.03	0.01	−0.03	0.03	0.06	0.49 ***	-		
9. Educational reconsideration of commitment	0.05	−0.01	0.02	0.06	0.03	−0.01	−0.26 **	−0.11 *	-	
10. School performance	−0.10 *	0.01	0.10 *	0.18 ***	−0.17 ***	−0.12 **	0.20 ***	0.19 ***	−0.15 **	-

Note: * *p* < 0.05, ** *p* < 0.01, *** *p* < 0.001.

**Table 3:** Differences in school experience according to the sleep-recovery strategies.

	Group Based on the Amount of Weekend Social Jetlag and Catch-Up Sleep					
	Social Jetlag < 90 min; Catch-Up Sleep < 60 min	Social Jetlag < 90 min; Catch-Up Sleep ≥ 60 min	Social Jetlag ≥ 90 min; Catch-Up Sleep < 60 min	Social Jetlag ≥ 90 min; Catch-Up Sleep ≥ 60 min	F (df)	η^2^
Educational commitment	3.18 (0.88)	3.31 (0.70)	3.21 (0.77)	3.28 (0.78)	F_3502_ = 0.65	0.00
Educational in-depth exploration	3.07 ^a^ (0.72)	3.37 ^b^ (0.66)	3.20 ^a,b^ (0.64)	3.27 ^a,b^ (0.68)	F_3502_ = 3.89 **	0.02
Educational reconsideration of commitment	2.90 (0.95)	2.96 (0.78)	3.05 (0.92)	2.96 (0.90)	F_3500_ = 0.56	0.00
School performance	7.24 (0.91)	7.50 (0.95)	7.20 (0.84)	7.30 (0.85)	F_3498_ = 2.34	0.01

Note: Mean and standard deviation in brackets are reported; ** *p* < 0.01; ^a^ mean is significantly different (*p* < 0.05) from another mean at the Tukey post hoc test if they have different superscripts.

## Data Availability

The data presented in this study are available on request from the corresponding author. The data are not publicly available due to ethical issues.

## Supplementary Materials

The following supporting information can be downloaded at https://www.mdpi.com/article/10.3390/brainsci13020178/s1, Table S1: Sleep characteristics of the four groups. Means (M) and standard deviations (SD) are shown.
