# Supplementary material for: The Association between Sleep Patterns, Educational Identity, and School Performance in Adolescents
Source: Brain Sci. 2023 Jan 20;13(2):178. doi: 10.3390/brainsci13020178 (PMC9954600; doi:10.3390/brainsci13020178)
Supplement: Supplementary file 1 [file brainsci-13-00178-s001.zip › brainsci-2145987-supplementary.pdf]

Table S1. Sleep characteristics of the four groups. Means (M) and standard deviations (SD) are shown.

**Sleep variables**	**Social Jetlag < 90 minutes; Catch-up sleep < 60 minutes** *M (SD)*	**Social jetlag < 90 minutes; Catch-up sleep ≥ 60 minutes** *M (SD)*	**Social jetlag ≥ 90 minutes; Catch-up sleep < 60 minutes** *M (SD)*	**Social jetlag ≥ 90 minutes; Catch-up sleep ≥ 60 minutes** *M (SD)*
Amount of social jetlag (In minutes)	42.58 (33.61)	52.30 (29.68)	151.72 (52.39)	145.21 (48.04)
Amount of catch-up sleep (In minutes)	-3.55 (50.19)	123.75 (64.26)	-17.10 (72.66)	141.20 (60.16)
Total sleep time during the week (In minutes)	419.60 (44.31)	396.43 (55.28)	415.27 (44.06)	391.17 (47.60)
Total sleep time during the weekend (In minutes)	416.05 (64.15)	520.18 (66.04)	398.17 (80.42)	532.54 (57.55)
